# mRNA and DNA Detection of Human Papillomaviruses in Women of All Ages Attending Two Colposcopy Clinics

**DOI:** 10.1371/journal.pone.0049205

**Published:** 2012-11-15

**Authors:** Aris Spathis, Christine Kottaridi, Aikaterini Chranioti, Christos Meristoudis, Charalambos Chrelias, Ioannis G. Panayiotides, Evangelos Paraskevaidis, Petros Karakitsos

**Affiliations:** 1 Department of Cytopathology, University General Hospital “ATTIKON”, School of Medicine, National and Kapodistrian University of Athens, Chaidari, Greece; 2 3^rd^ Department of Obstetrics and Gynecology, University General Hospital “ATTIKON”, School of Medicine, National and Kapodistrian University of Athens, Chaidari, Greece; 3 2^nd^ Department of Pathology, University General Hospital “ATTIKON”, School of Medicine, National and Kapodistrian University of Athens, Chaidari, Greece; 4 Department of Obstetrics and Gynecology, University Hospital of Ioannina, Ioannina, Greece; The Chinese University of Hong Kong, Hong Kong

## Abstract

**Objective:**

HPV infection is a common finding, especially in young women while the majority of infections are cleared within a short time interval. The aim of this study was to examine the efficacy of HPV DNA and mRNA testing in a population attending colposcopy units of two University hospitals.

**Methods:**

1173 liquid based cervical samples from two colposcopy clinics were tested for HPV DNA positivity using a commercial typing kit and HPV E6/E7 mRNA positivity with a flow cytometry based commercial kit. Statistic measures were calculated for both molecular tests and morphological cytology and colposcopy diagnosis according to histology results.

**Results:**

HPV DNA, high-risk HPV DNA, HPV16 or 18 DNA and HPV mRNA was detected in 55.5%, 50.6%, 20.1% and 29.7% of the cervical smears respectively. Concordance between the DNA and the mRNA test was 71.6% with their differences being statistically significant. Both tests’ positivity increased significantly as lesion grade progressed and both displayed higher positivity rates in samples from women under 30 years old. mRNA testing displayed similar NPV, slightly lower sensitivity but significantly higher specificity and PPV than DNA testing, except only when DNA positivity for either HPV16 or 18 was used.

**Conclusions:**

Overall mRNA testing displayed higher clinical efficacy than DNA testing, either when used as a reflex test or as an ancillary test combined with morphology. Due to enhanced specificity of mRNA testing and its comparable sensitivity in ages under 25 or 30 years old, induction of mRNA testing in young women could be feasible if a randomized trial verifies these results.

## Introduction

Human Papillomavirus (HPV) infection is estimated to be the most common sexually transmitted infection [1] and has been directly linked to cervical intraepithelial lesion creation and increased risk of cervical cancer development [2], [3]. Although HPVs are commonly detected in cervical smears, most infections appear to be transient and asymptomatic, with approximately 90% of infections being cleared within 2 years [4], [5], [6]. Only persistent HPV infections seem to be linked to CIN establishment and progression [5], [7], [8].

Although the life-long risk for HPV infection is 80%, only a fraction of that percentage of women will eventually develop cervical cancer [4], [9]. Furthermore, HPV DNA positivity usually peaks in younger women, ranging from 25%–65% with a steady decline in women older than 35 years, ranging from 10%–30% [4], [6], [10], [11]. As a result HPV DNA testing has been suggested for screening of women over 30 or women with equivocal cytology results [12], [13].

The expression of the two viral genes E6 and E7 is responsible for the transformation of cells by HPV, resulting in continuous morphological changes and finally cervical neoplasia [14], [15], [16], [17], [18]. Therefore, detection of HPV oncogene transcripts has been proposed to identify more accurately women with higher grade lesions and increased risk of cervical cancer development [19], [20], [21], [22], [23].

In the present study the clinical performance of flow cytometric in-situ hybridization for E6 and E7 mRNA transcript detection was evaluated, in women attending two colposcopy clinics of two University Hospitals in Greece.

## Materials and Methods

### Study Design

A total of 1173 women that attended the colposcopy clinics of the 3^rd^ Department of Gynecology and Obstetrics in the tertiary care “ATTIKON” University General Hospital and the Department of Gynecology and Obstetrics in the University Hospital of Ioannina were enrolled in the present study. The study population did not represent a normally screened population, since most patients attended the outpatient clinics after a referral abnormal cytology and or colposcopy. All patients signed an informed consent form, while the study was approved by the Bioethics comity of the hospitals. A liquid-based cytology (LBC, ThinPrep® Pap-Test, Hologic, U.S.A.) sample was collected and a monolayer smear was prepared on a TP 2000 Processor and stained according to Papanicolaou technique. A trained cytopathologist diagnosed each case, according to the Bethesda 2001 system [24]. Biopsies were taken from all women with either colposcopically visible lesions or a cytology result of ASCUS or worse. Tissue samples were routinely processed and diagnosed by an experienced pathologist. Two 1 ml LBC aliquots were used for DNA extraction and flow cytometric analysis.

### HPV DNA Detection

**Figure 1:**
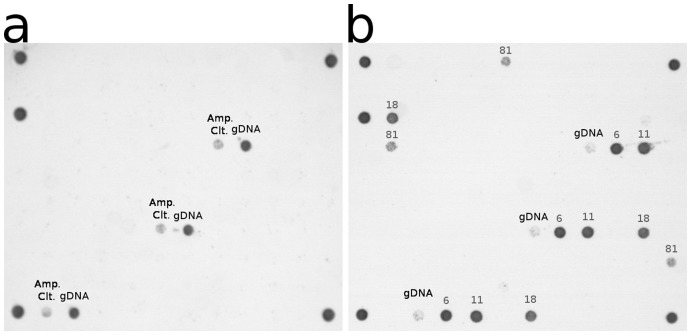
Array tube image of a CLART® HPV 2 negative clinical sample (a) and a HPV positive sample with multiple HPV types present (b). Both the amplification and the genomic DNA control are present in the negative sample (a), while loss of the amplification control is common in HPV positive samples.

HPV DNA detection was performed on the first aliquot using a commercially available kit, CLART® HPV 2 kit (Genomica, Spain) that allowed the detection of 35 different HPV genotypes, 20 HR-HPVs (16, 18, 26, 31, 33, 35, 39, 45, 51, 52, 53, 56, 58, 59, 66, 68, 70, 73, 82 & 85) and 15 LR-HPVs (6, 11, 40, 42, 43, 44, 54, 61, 62, 71, 72, 81, 83, 84 & 89) as categorized by Dunne et al. [11] CLART uses biotinylated primers (PGMY09/11) that amplify a 450 bp fragment of the HPV L1 locus. Co-amplification of an 892 bp region of the CFTR gene and a 1,202 bp fragment of a transformed plasmid provide positive controls for DNA extraction adequacy and PCR efficiency. Amplicons were detected via hybridization in a low-density microarray containing triplicate DNA probes for the 35 HPV types and controls (Figure 1).

### Flow Cytometric E6/E7 HPV mRNA Detection

**Figure 2:**
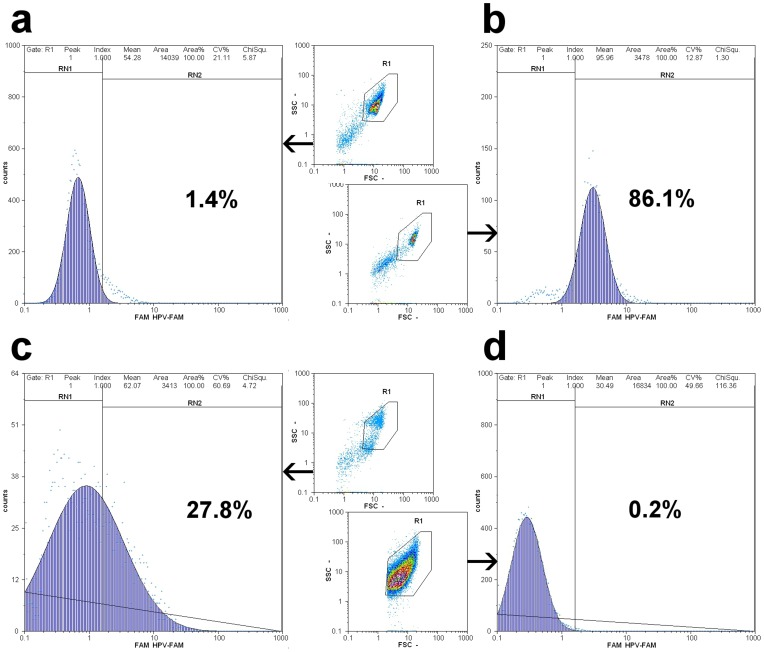
Oncotect™ E6/E7 mRNA results of a) HPV Negative control cells, b) HPV Positive control cells, c) a CIN2 sample and d) a clinical negative sample. The population of interest is gated (R1) on a green fluorescence/count histogram. Positive samples contain >1.5% of cells in the cut-off gate (RN2).

The other 1 ml aliquot of the LBC specimen was used for whole cell detection of E6/E7 HPV mRNA over-expression by means of a commercially available kit (HPV Oncotect™ E6/E7 mRNA kit, IncellDx, CA, U.S.A.). Briefly, cells were washed in PBS to remove the preservative, fixed for 1 h at room temperature and washed with two pre-hybridization buffers. HPV mRNA was labeled after a 30′ hybridization at 43°C with a FAM labeled probe cocktail, followed by two stringency washes of the cells in order to remove the unbound probe. Cells were resuspended in 1 ml PBS containing 2% fetal calf and analysed on a Partec CyFlow SL with a 488 nm argon laser, with front-scatter (FSS) and side-scatter (SSC) set on logarithmic scale. Cells of interest were gated as previously described, and the cut-off for positive samples was set 1.5% of green fluorescent cells. Two cell lines commercially obtained by IncellDx (HPV Positive control Cells and HPV Negative Control Cells) were used to verify gating of cells and the cut-off threshold (Figure 2).

### Data Analysis

Both morphology and molecular results were independently collected and both pathologists and biologist were blinded from the results of the other part. Statistical analysis of the data was performed using SPSS19 (IBM coorporation, U.S.A.). Analysis of results was performed using fishers exact test for 2 by 2 tables and χ^2^ for trend for other tables. Comparison of results of the two molecular tests was performed using the McNemar test and their agreement was accessed using the κ value. Comparison of molecular testing with cytology was performed using the McNemar test in CIN2- for specificity and in CIN2+ for sensitivity. Sensitivity, specificity, positive and negative predictive values (PPV, NPV) and odds ratios were calculated using a 95% confidence interval. Finally correlations between assessed parameters were calculated using the Spearman test.

## Results

### Morphology Results

Study participants were between ages of 19–81 years with a mean of 38.2 years of age. Out of 1173 women 538 had a LSIL+ colposcopy result and 6 women with referral cytology of HSIL+ had a non-satisfactory colposcopy result (Table 1). 53 women with negative colposcopy had a cytology result of ASCUS+. In all these cases punch biopsies were taken raising the number of histological verified samples to 597 (50.8%). The remaining 576 samples had negative results in both cytology and colposcopy and were considered clinically negative. Colposcopy and cytology, as expected, displayed high correlation with histology with spearman values of 0.893 and 0.875 respectively (Table 1).

**Table 1:** Morphology results with histology findings.

		Histology Result							
		N/A	Negative	CIN1	CIN2	CIN3	SCC	AIS	Total
**Colposcopy Result**	NSF	0	3 (2.2%)	3 (1.1%)	0	0	0	0	**6 (0.5%)**
	Negative	576 (100%)	29 (20.9%)	19 (7.3%)	3 (3.3%)	0	0	2 (18.2%)	**629 (53.6%)**
	LSIL	0	97 (69.8%)	211 (80.8%)	38 (42.2%)	12 (15.4%)	0	1 (9.1%)	**359 (30.6%)**
	HSIL	0	10 (7.2%)	28 (10.7%)	49 (54.4%)	65 (83.3%)	4 (22.2%)	1 (9.1%)	**157 (13.4%)**
	SCC	0	0	0	0	1 (1.3%)	13 (72.2%)	0	**14 (1.2%)**
	AIS	0	0	0	0	0	1 (5.6%)	7 (63.6%)	**8 (0.7%)**
**Cytology Result**	Inadequate	0	3 (2.2%)	0	0	0	0	0	**3 (0.3%)**
	NILM	576 (100%)	49 (35.3%)	30 (11.5%)	4 (4.4%)	2 (2.6%)	0	0	**661 (56.4%)**
	ASC-US	0	31 (22.3%)	69 (26.4%)	10 (11.1%)	3 (3.8%)	0	1 (9.1%)	**114 (9.7%)**
	LSIL	0	42 (30.2%)	141 (54%)	26 (28.9%)	8 (10.3%)	0	1 (9.1%)	**218 (18.6%)**
	ASC-H	0	5 (3.6%)	2 (0.8%)	1 (1.1%)	1 (1.3%)	1 (5.6%)	0	**10 (0.9%)**
	HSIL	0	9 (6.5%)	19 (7.3%)	49 (54.4%)	64 (82.1%)	8 (44.4%)	2 (18.2%)	**151 (12.9%)**
	SCC	0	0	0	0	0	8 (44.4%)	1 (9.1%)	**9 (0.8%)**
	AIS	0	0	0	0	0	1 (5.6%)	6 (54.5%)	**7 (0.6%)**
**Total**		**576 (49.1%)**	**139 (11.8%)**	**261 (22.3%)**	**90 (7.6%)**	**78 (6.6%)**	**18 (1.5%)**	**11 (0.9%)**	**1173**

N/A: Not available, NSF: Non satisfactory, LSIL: Low grade intra-epithelial lesion, HSIL: High grade intra-epithelial lesion, SCC: Squamous cell carcinoma, CIN: cervical intraepithelial neoplasia, ASC-US: Atypical squamous cells of unknown significance, ASC-H: Atypical squamous cells cannot exclude high grade, AIS: Adenocarcinoma in Situ.

### Molecular Results

Results of molecular testing according to histology findings are summarized in table 2. Overall positivity for either HPV mRNA or DNA was significantly higher as lesion grade progressed (p<0.001, Table 2). HPV DNA positivity was, as expected, high in clinically negative cases (40.5%) and rose at 92% in CIN2+ cases. For high risk HPV DNA similar results were observed (36.1% for negative and 90% for CIN2+), while for HPV16 or 18 positivity was significantly lower (8.5% for negative and 58% for CIN2+). mRNA positivity was lower for clinically and histologically negative samples (14.2% and 12.2%), doubled in CIN1 samples (30.7%) and rose to 86% in CIN2+ cases. All tests’ positivity rates increased in a statistically significant percent in CIN1+, CIN2+, CIN3+ categories with different correlation co-efficiency values ranging from 0.433, 0.552 and 0.397 for mRNA in the three categories, to 0.358, 0.333 and 0.248 for DNA in the same categories.

**Table 2:** Molecular results with histology findings.

		Histology Result							
		N/A	Negative	CIN1	CIN2	CIN3	SCC	AIS	Total
CLART2Result	Inadequate	7 (1.2%)	1 (0.7%)	6 (2.3%)	0	0	0	0	14 (1.2%)
	Negative	336 (58.3%)	76 (54.7%)	81 (31%)	9 (10%)	3 (3.8%)	2 (11.1%)	1 (9.1%)	508 (43.3%)
	Positive	233 (40.5%)	62 (44.6%)	174 (66.7%)	81 (90%)	75 (96.2%)	16 (88.9%)	10 (90.9%)	651 (55.5%)
	Positive for HR	208 (36.1%)	56 (40.3%)	152 (58.2%)	78 (86.7%)	75 (96.2%)	14 (77.8%)	10 (90.9%)	593 (50.6%)
	Positive for HPVs 16 or 18	49 (8.5%)	18 (12.9%)	59 (22.6%)	37 (41.1%)	55 (70.5%)	12 (66.7%)	6 (54.5%)	236 (20.1%)
IncellDxResult	Inadequate	5 (0.9%)	4 (2.9%)	14 (5.4%)	7 (7.8%)	4 (5.1%)	0	2 (18.2%)	36 (3.1%)
	Negative	489 (84.9%)	118 (84.9%)	167 (64.0%)	7 (7.8%)	6 (7.7%)	2 (11.1%)	0	789 (67.3%)
	Positive	82 (14.2%)	17 (12.2%)	80 (30.7%)	76 (84.4%)	68 (88.9%)	16 (88.9%)	9 (81.8%)	348 (29.7%)
Total		576 (49.1%)	139 (11.8%)	261 (22.3%)	90 (7.7%)	78 (6.6%)	18 (1.5%)	11 (0.9%)	1173

mRNA and DNA testing showed significantly different results as depicted in Table 3. IncellDx mRNA positivity was almost half of CLART2 DNA positivity (29.7% vs. 55.5, p<0.001), significantly lower than HR DNA positivity (50.6%, p<0.001) and higher than HPV16 or 18 DNA positivity (20.1%, p<0.001). Overall agreement of DNA and mRNA testing was good (62.3–73.8%). 57 HPV DNA negative and 71 HR HPV DNA samples were mRNA positive. Furthermore, mRNA had significantly more inadequate samples (36 vs. 14, p = 0.007).

**Table 3:** Molecular results concordance.

		IncellDx Result						
		Inadequate	Negative	Positive	Agreement	McNemar	κ	Total
CLART2 Result	Inadequate	3 (8.3%)	9 (1.1%)	2 (0.6%)				14 (1.2%)
	Negative	12 (33.3%)	439 (55.6%)	57 (16.4%)				508 (43.3%)
	Positive	21 (58.3%)	341 (43.2%)	289 (83.0%)				651 (55.5%)
	Positive for HR	18 (50.0%)	298 (37.8%)	277 (79.6%)	762 (64.9%)	<0.001	0.333	593 (50.6%)
	Positive for HPVs16 or 18	7 (19.4%)	73 (9.3%)	156 (44.8%)	866 (73.8%)	<0.001	0.363	236 (20.1%)
Total		36 (3.1%)	789 (67.2%)	348 (29.7%)	731 (62.3%)	<0.001	0.307	1173

### Clinical Efficiency

Clinical efficiency of morphology and molecular tests was evaluated with classic statistic measures using as golden standard histology results (Table 4). Morphology tests have a bias over molecular testing since the clinically negative population is based on cytology and colposcopy with no histology result and were mainly analyzed as standards to compare molecular testing results. As expected, cytology displayed the highest sensitivity and negative predictive value (NPV) using ASCUS+ as cut-off and the highest specificity, positive predictive value (PPV) and odds ratio using ASCH+ as cut-off. Similar results were observed for colposcopy using LSIL and HSIL as cut-offs.

**Table 4:** Clinical efficiency of morphology and molecular tests.

Histology endpoint CIN2+	Sensitivity (TP)	Specificity (TN)	PPV	NPV	Odds ratio	Recalls (TP+FP)	Missed % of CIN2+ (FN)
**ASCUS+ cytology**	97.0 (191)	67.4 (658)	37.5	99.1	65.7	43.3% (509)	3.0% (6)
**ASCUS+ cytology without clinical negative**	97.0 (191)	20.5 (82)	37.5	93.2	8.2	85.2% (509)	3.0% (6)
**ASCH+ cytology**	72.1 (142)	96.4 (941)	80.2	94.5	69.4	15.0% (177)	27.9% (55)
**ASCH+ cytology without clinical negative**	72.1 (142)	91.3 (365)	80.2	86.9	26.9	29.6% (177)	27.9% (55)
**LSIL+ colposcopy**	97.5 (192)	64.5 (630)	35.7	99.2	69.9	45.8% (538)	2.5% (5)
**HSIL+ colposcopy**	71.6 (141)	96.1 (938)	78.8	94.4	62.1	15.2% (179)	28.4% (56)
**CLART2**	92.4 (182)	51.9 (507)	27.9	97.1	13.1	55.5% (651)	7.6% (15)
**CLART2 HR**	89.8 (177)	57.4 (560)	29.8	96.6	11.9	50.5% (593)	10.2% (20)
**CLART2 HPV16 or 18**	55.8 (110)	87.1 (850)	46.6	90.7	8.5	20.1% (236)	44.2% (87)
**IncellDx**	85.8 (169)	81.6 (797)	48.6	96.6	26.8	29.6% (348)	14.2% (28)
**ASCUS+ CLART+**	89.8 (177)	79.3 (774)	46.7	97.5	33.9	32.2% (379)	10.2% (20)
**ASCUS+ IncellDx+**	83.8 (165)	90.8 (886)	64.7	96.5	50.7	21.7% (255)	16.2% (32)
**ASCH+ or ASCH− CLART+**	97.5 (192)	50.7 (495)	28.5	99.0	39.5	57.4% (673)	2.5% (5)
**ASCH+ or ASCH− IncellDx+**	95.9 (189)	79.8 (779)	49.0	99.0	93.4	32.9% (386)	4.0% (8)
**ASCH+ or ASCUS/LSIL/CLART+**	94.9 (187)	77.9 (760)	46.4	98.7	65.8	34.3% (403)	5.0% (10)
**ASCH+ or ASCUS/LSIL/IncellDx +**	93.9 (185)	88.9 (868)	63.1	98.6	123.9	24.9% (293)	6.1% (12)

Statistical measures have been calculated using 197 CIN2+, 400 histological confirmed CIN2- and 576 clinically negative cases, unless otherwise stated. Inadequate or invalid molecular testing results have been considered as negative and have not been excluded in order to measure clinical efficiency of the tests in every day conditions. TP: True positive, FN: False negative, TN: True Negative, FP: False positive.

CLART displayed higher positivity rates in CIN2+ cases that mRNA (182 vs. 169, p = 0.044), as mirrored by higher sensitivity values (Table 2). This difference was not significant in CLART HR positive CIN2+ (177 vs. 169, p = 0.17) and was inverted in CLART HPV16 18 (110 vs. 169, p = 0.15). However both specificities, PPVs and odds ratios was significantly higher for mRNA in all categories analyzed (Table 4). NPVs were similar for both tests.

Combination of molecular testing and cytology was analyzed using three distinct scenarios (Table 4). These included using either molecular tests as a reflex test for ASCUS+ or ASCH- and finally referring all ASCH+ and molecular testing positive cases of ASCUS and LSIL. The last was the best combination doubling odds ratio for ASCH+ alone and increasing 5 times the odds ratio of IncellDx alone. Combination of DNA testing to cytology increased CLARTs statistic measures without them surpassing cytology alone.

### Age Dependent Efficiency

**Figure 3:**
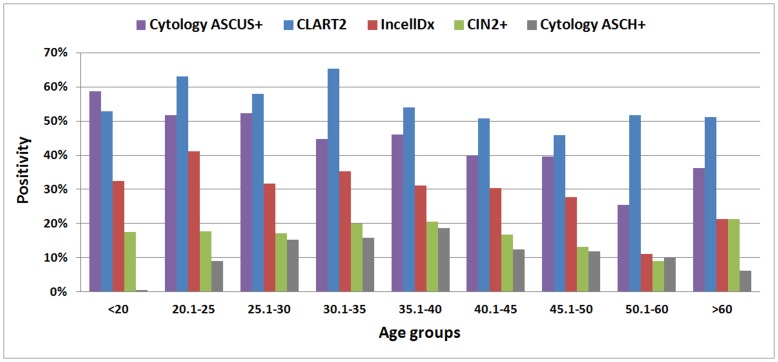
Positivity rates in different age groups.

The study population was divided in 9 age groups with the two extreme categories (<20 and >60) having the least cases and all other containing about the same number of samples. Positivity for morphology and molecular tests peaked at different age groups (Figure 3). DNA positivity peaked at 20–25 and 30–35 with a decline over 35 years of age, while mRNA positivity peaked at 20–25 and followed a similar profile. CIN2+ positivity was highest in ages >60 with a second peak in ages 30–40, while ASCUS+ cases had a decline according to age, a profile opposite to ASCH+ cases that displayed an increase with age up to the age of 40 and a decline after on. CIN1+ cases were significantly more often in the specific population in women under 30 (49.1% vs. 34.9%, p<0.001) without reaching significance in <25 years of age (p = 0.105). CIN2+ cases were equally distributed using as cut-off either 25 or 30 years of age, while CIN3+ were significantly more in both >25 and >30 age groups (p = 0.017 and p = 0.015). Even though CIN2+ cases had similar distribution in the two age groups of over and under 25, cytopathologists tended to underestimate the lesions in women under 25. This is reflected by the reduced percentage of CIN2+ cases that were diagnosed as ASCH+ in women under 25 (11/24, 45%) vs. women over 25 (131/173, 75%) that was statistically different (p = 0.006).

Comparison of molecular testing efficiency was performed in dichotomized groups with cut-off either 25 or 30 years of age (Table 5). The most sensitive marker was CLART, while HR CLART positivity and IncellDx showed comparable results and positivity for HPV16 or 18 had the lowest sensitivity. On the other hand specificity and PPV were significantly higher for IncellDx and HPV16/18, while NPV was similar for all tests. Overall mRNA performed better that DNA testing as reflected by the odds ratios. Positivity correlated with lesion progression with a significance of <0.001 in all tests, except for DNA positivity in women <25 in the CIN3+ category (p = 0.03).

**Table 5:** Clinical efficiency of molecular tests in different age groups.

Histology endpoint CIN2+	Age	Number	Sensitivity(TP)	Specificity(TN)	PPV	NPV	Odds ratio	Recalls (TP+FP)	Missed % of CIN2+ (FN)
**CLART2**	<25	140	87.5 (21)	49.1 (57)	26.3	95.0	6.7	57.1% (80)	12.5% (3)
	>25	1033	93.1 (161)	52.3 (450)	28.2	97.4	14.7	55.3% (571)	6.9% (12)
	<30	340	95.1 (58)	47.0 (131)	28.2	97.8	17.1	60.6% (206)	4.9% (3)
	>30	833	81.2 (124)	53.9 (376)	27.9	96.9	12.1	53.4% (445)	12.5% (12)
**CLART2 HR**	<25	140	87.5 (21)	57.8 (67)	30.0	95.7	9.5	50.0% (70)	12.5% (3)
	>25	1033	90.2 (156)	57.3 (493)	29.8	96.7	12.3	50.6% (523)	9.8% (17)
	<30	340	91.8 (56)	54.1 (151)	30.4	96.8	13.2	54.1% (184)	8.2% (5)
	>30	833	89.0 (121)	58.7 (409)	29.6	96.5	11.4	49.1% (409)	11.0% (15)
**CLART2 HPV16** **or 18**	<25	140	58.3 (14)	86.2 (100)	46.7	90.9	8.7	21.4% (30)	41.7% (10)
	>25	1033	55.5 (96)	87.2 (750)	46.6	90.7	8.5	19.9% (206)	44.5% (77)
	<30	340	62.3 (38)	84.2 (235)	46.3	91.1	8.8	24.1% (82)	37.7% (23)
	>30	833	52.9 (72)	88.8 (615)	46.8	90.6	8.4	18.5% (154)	47.1% (64)
**IncellDx**	<25	140	91.7 (22)	75.0 (87)	43.1	97.8	33	36.4% (51)	8.3% (2)
	>25	1033	85.0 (147)	82.6 (710)	49.5	96.5	26.7	28.8% (297)	15% (26)
	<30	340	90.2 (55)	76.6 (213)	45.5	97.3	29.6	35.6% (121)	9.8% (6)
	>30	833	83.8 (114)	83.8 (534)	50.2	96.4	26.8	33.3% (277)	16.2% (22)
**Cytology ASCUS+**	<25	140	95.8 (23)	57.8 (67)	31.9	98.5	31.5	51.4% (72)	4.2% (1)
	>25	1033	97.1 (168)	68.7 (591)	38.4	99.2	73.8	42.3% (437)	2.9% (5)
	<30	340	96.7 (59)	55.2 (154)	32.1	98.7	36.3	54.2% (184)	3.3% (2)
	>30	833	97.1 (132)	72.3 (504)	40.6	99.2	86.1	39% (325)	2.9% (4)

Statistical measures have been calculated using 197 CIN2+, 400 histological confirmed CIN2- and 576 clinically negative cases. Inadequate or invalid molecular testing results have been considered as negative and have not been excluded in order to measure clinical efficiency of the tests in every day conditions. TP: True positive, FN: False negative, TN: True Negative, FP: False positive.

Positivity was higher in women under 30 years of age for IncellDx, CLART and CLART HPV16/18 (p = 0.005, p = 0.023, p = 0.031) while the same result didn’t reach statistical significant in ages under 25. In CIN3+ lesions positivity rate for HPV16/18 in women under 30 was statistically higher than in CIN3+ cases of women over 30 (90.5% vs. 62.8%, p = 0.015). Positive mRNA samples were statistically more often CIN3+ in women over 25 or over 30 than in younger women (29.3% vs. 11.8%, p = 0.005 and 32.6% vs. 15.7%, p<0.001). The same was observed for HPV DNA and HR HPV DNA with weaker strength of association (p = 0.011, p = 0.021). HR DNA or HPV16/18 positivity was more often a CIN1+ in women under 30 (67.9% vs. 47.9% and 81.7% vs. 66.2, p<0.001).

### Molecular Testing Versus Cytology Efficiency

Even though, there was an obvious bias for cytology due to the clinically negative cases, a sub-analysis was performed comparing ASCUS+ cytology diagnosis to molecular testing, triggered by the observation that CIN2+ cases were correlated with higher spearman values to mRNA testing (0.552) than ASCUS + cytology (0.486). This difference was more profound when analysing only histological confirmed cases (0.582 for mRNA vs. 0.232 for ASCUS+). DNA testing had lower correlation co-efficiencies than ASCUS + ranging from 0.33 to 0.40.

ASCUS+ displayed higher sensitivity in CIN2+ cases than HPV DNA (McNemar p = 0.064), HR HPV DNA (p = 0.007), HPV16/18 DNA (p<0.001) and mRNA (p<0.001) (Tables 5 and 6). On the other hand specificity was significantly higher for HPV16/18 DNA and mRNA (p<0.001) whether clinically negative cases were included or not, while for HPV DNA and HR HPV DNA specificity was significantly higher (p<0.001) only when clinically negative samples were omitted.

**Table 6:** Molecular tests versus cytology.

CIN2- (N = 976)	Age (N)	ASCUS– (N = 658)	ASCUS+ (N = 318)	McNemar	CIN2+ (N = 197)	Age	ASCUS– (N = 6)	ASCUS+ (N = 191)	McNemar
**CLART2+**	<25 (116)	25	34	0.154	**CLART2+**	<25 (24)	1	20	0.657
	>25 (860)	242	168	0.002		>25 (173)	4	157	0.118
	<30 (279)	57	91	0.21		<30 (61)	2	56	1.0
	>30 (697)	210	111	<0.001		>30 (136)	3	121	0.057
	All (976)	267	202	<0.001		All (197)	5	177	0.064
**CLART2 HR+**	<25 (116)	1	28	1.0	**CLART2 HR+**	<25 (24)	1	20	0.625
	>25 (860)	28	151	<0.001		>25 (173)	4	152	0.012
	<30 (279)	48	80	0.836		<30 (61)	2	54	0.453
	>30 (697)	189	99	<0.001		>30 (136)	3	118	0.013
	All (976)	237	179	<0.001		All (197)	5	172	0.007
**CLART2 HPV16 or 18+**	<25 (116)	0	13	<0.001	**CLART2 HPV16 or 18+**	<25 (24)	1	13	0.012
	>25 (860)	8	56	<0.001		>25 (173)	2	94	<0.001
	<30 (279)	10	34	<0.001		<30 (61)	1	37	<0.001
	>30 (697)	47	35	<0.001		>30 (136)	2	70	<0.001
	All (976)	57	69	<0.001		All (197)	3	107	<0.001
**IncellDx +**	<25 (116)	1	13	<0.001	**IncellDx +**	<25 (24)	1	21	1.0
	>25 (860)	6	77	<0.001		>25 (173)	3	144	<0.001
	<30 (279)	31	35	<0.001		<30 (61)	1	54	0.219
	>30 (697)	58	55	<0.001		>30 (136)	3	111	<0.001
	All (976)	89	90	<0.001		All (197)	4	165	<0.001
**ASCH+ or ASCUS/LSIL/CLART+**	<25 (116)	0	34	<0.001	**ASCH+ or ASCUS/LSIL/CLART+**	<25 (24)	0	22	1.0
	>25 (860)	0	180	<0.001		>25 (173)	0	165	0.250
	<30 (279)	0	91	<0.001		<30 (61)	0	58	1.0
	>30 (697)	0	123	<0.001		>30 (136)	0	129	0.250
	All (976)	0	214	<0.001		All (197)	0	187	0.125
**ASCH+ or ASCUS/LSIL/IncellDx+**	<25 (116)	0	13	<0.001	**ASCH+ or ASCUS/LSIL/IncellDx+**	<25 (24)	0	22	1.0
	>25 (860)	0	95	<0.001		>25 (173)	0	163	0.063
	<30 (279)	0	38	<0.001		<30 (61)	0	57	0.500
	>30 (697)	0	70	<0.001		>30 (136)	0	128	0.125
	All (976)	0	108	<0.001		All (197)	0	185	0.031

Statistical measures have been calculated using 197 CIN2+, 400 histological confirmed CIN2- and 576 clinically negative cases. Inadequate or invalid molecular testing results have been considered as negative and have not been excluded in order to measure clinical efficiency of the tests in every day conditions.

By including the age variable in the analysis, significant differences of sensitivity and specificity were identified in the different age groups (Tables 5 and 6). Both HPV DNA and HR HPV DNA positivity displayed slightly lower sensitivity than ASCUS+ cytology with significantly lower specificity in older ages. On the other hand both mRNA and HPV16/18 positivity had significantly higher specificity than ASCUS+ cytology, but only mRNA testing had comparable sensitivity in ages under 25 or 30 years of age. In older women sensitivity of ASCUS+ was significantly higher. Combination of cytology of ASCH+ with either DNA or mRNA positivity in ASCUS+ or LSIL+ displayed significantly higher specificity and comparable specificity in all age groups examined.

## Discussion

In the present study 1173 samples from women visiting colposcopy units of two tertiary hospitals in Greece were used to identify molecular testing efficiency. Most studies designed to evaluate efficacy of molecular testing, are large scale randomized screening trials, while retrospective analysis of histological confirmed cases has greatly aided in connecting HPV with cervical neoplasia [13], [19], [25], [26]. It is clearly stated that the population selected did not represent a normally screened population. However there was no bias in patient selection apart from their willingness to participate after signing an informed consent form. In order to have adequate data for analysis a significant number of samples with abnormalities was needed that was provided from colposcopy clinics at which women with previously positive pap results attend. The only bias of this study lies in morphology efficiency results since the clinically negative cases had all negative cytology and colposcopy, a fact that is reflected on significant changes of their NPVs and specificities after exclusion of clinical negative samples.

The study population included many samples with abnormalities of either low or high grade and an increased percentage of carcinomas. Molecular testing had increased positivity as lesion grade progressed, as expected. However mRNA positivity was significantly lower, especially in clinically negative, histological negative and CIN1 samples, in concordance with previously studies [19], [20], [22]. This result was expected since HPV DNA testing identifies infections that can be transient with no clinical significance, while mRNA detection of E6/E7 transcripts reflect, due to their biologic role, an infection that could be persistent and lead to lesion progression [5], [27]. DNA and mRNA positivity was not significantly different between clinical negative and histologically negative samples, suggesting that these represent, in a significant percent, true negative samples.

Since both colposcopy clinics have experienced colposcopists and all cytologic diagnoses were set by experienced cytopathologists, morphology yielded excellent results with highest sensitivities and NPVs calculated when using as cut-offs ASCUS for cytology and LSIL for colposcopy and highest specificities and PPVs for ASCH+ cytology and HSIL+ colposcopy. mRNA HPV displayed characteristics between the two cut-offs with higher PPV than ASCUS+ and higher sensitivity than ASCH+. On the other hand DNA testing displayed much worse specificity apart from when considering presence of HPV16 or HPV18. It has been previously shown that mRNA testing has significantly higher specificity and PPV than DNA testing [19], either when compared with typing methods [20] or HC2 [21], [22]. NASBA as a alternative mRNA testing has also shown significant differences from DNA testing especially for HPV 16 [23]. NASBA has shown such high statistic measures that it has been proposed as alternative to repeat cytology in LSIL cases [28]. However NASBA is suggested to amplify also HPV DNA in high viral loads [29] that would result, in theory at least, in increased sensitivity. Carrying into mind that DNA detection of HPV16 or HPV18 has increased specificity for CIN2+, coamplification of DNA with NASBA wouldn’t impair NASBAs specificity values.

More recently, many have either proposed replacement of cytology with molecular testing for either HPV DNA or HPV RNA, or their combined use is specific categories of lesions. For instance, it has been shown that combination of LSIL+ cytology and HPV DNA cases positive for HPV16/18 in women over 25 displayed significantly higher sensitivity and similar PPV that ASCUS+ cytology alone [13]. Others found no difference in combining cytology and mRNA [28]. In our population combination of cytology with either molecular testing would improve the performance of the test. However, only when mRNA testing was used in combination with cytology, the results were significantly better that cytology alone, as reflected by the high increase of odds ratio. Combination of cytology of ASCH+ with either DNA or mRNA positivity in ASCUS+ or LSIL+ displayed significantly higher specificity and comparable sensitivity to ASCUS+ cytology in all age groups examined. However, since there is a bias for cytology these results could change significantly in favor of molecular tests should a randomized trial be organized.

Since HPV positivity has been shown to peak in ages under 30 years old with a steady decline in older women [11] and women under 25 years clear the infection within 1 year after the detection [4], [6], HPV DNA testing was initially only proposed in women over years of age [30]. However excluding women younger than 30 from DNA testing would reduce the percentage of CIN2+ detected, as it has been reported that 35% of CIN2 would be missed [31]. In our study population, cytology with an ASCH+ for treatment would have missed 13 CIN2+ in women under 25 (54.1%) and 30 in women under 30 (43.4%).

Even though the population of the study was not a screening population, HPV DNA positivity followed the same profile with its peak in ages 20.1–25 years of age but with a second peak at 30.1–35 years of age. The same profile was followed by mRNA positivity. On the other hand only CIN1+ cases were statistically more often in ages under 30, while CIN2+ cases were similar for both age groups, CIN+3 cases were more often in women over 30 and cancers were only present in the group of women over 30. Surprisingly, mRNA results were better for the under 25 years of age group, an observation that could be result of the limited number of samples (n = 140). As far as HPV DNA is concerned CIN3+ cases of women under 30 years of age had significantly higher positivity for HPV16 or 18, suggesting that these two types are the most important types for lesion progression in younger women and that type specific DNA testing for these women could have potential benefits.

In sum, mRNA testing for E6/E7 outperformed DNA testing either when used alone in younger ages or overall in combination with cytology results. Since the study population originated from a colposcopy clinic, a larger scale screening population is needed to verify mRNA testing’s predominance. Furthermore, a longitual study with timed follow-up of patients is necessary in order to track the behavior of cytology negative samples with positive mRNA tests.
